# Tuberculosis Detection from Cough Recordings Using Bag-of-Words Classifiers

**DOI:** 10.3390/s25196133

**Published:** 2025-10-03

**Authors:** Irina Pavel, Iulian B. Ciocoiu

**Affiliations:** Faculty of Electronics, Telecommunications and Information Technology, Gheorghe Asachi Technical University of Iasi, Bd. Carol I 11A, 700050 Iasi, Romania; irina.pavel@etti.tuiasi.ro

**Keywords:** tuberculosis, cough, bag-of-words

## Abstract

The paper proposes the use of Bag-of-Words classifiers for the reliable detection of tuberculosis infection from cough recordings. The effect of using both independent and combined distinct feature extraction procedures and encoding strategies is evaluated in terms of standard performance metrics such as the Area Under Curve (AUC), accuracy, sensitivity, and F1-score. Experiments were conducted on two distinct large datasets, using both the original recordings and extended versions obtained by augmentation techniques. Performances were assessed by repeated *k*-fold cross-validation and by employing external datasets. An extensive ablation study revealed that the proposed approach yields up to 0.77 accuracy and 0.84 AUC values, comparing favorably against existing solutions and exhibiting robustness against various combinations of the setup parameters.

## 1. Introduction

What do Vivien Leigh, Frédéric Chopin, D. H. Lawrence, and Erwin Schrödinger have in common? Well, those are only some of the names of famous people who died from tuberculosis (TB). This contagious, infectious disease is caused by the pathogenic bacteria *Mycobacterium tuberculosis*, which is supposed to have appeared more than 150 million years ago [1]. Its presence has been evidenced even in Egyptian mummies, and documented in written Indian and Chinese texts more than 2000 years before [2,3]. Excellent, highly informative papers surveyed the turbulent history of this terrible plague [4,5], which is responsible for a death toll of about 1.25 million people per year even nowadays [6]. The texts present the successive contributions of many people aiming at revealing the cause of the disease, culminating with Robert Koch’s historic presentation on the etiology of tuberculosis from March 24, 1882, to the Berlin Physiological Society [7].

While TB can be prevented and cured by current-day therapies, the treatment may last for many months and face multidrug-resistant variants of the disease. Moreover, since the TB germs are spread through the air (by cough, sneezing, or speech), they typically affect the lungs, but other organs can also become infected (extrapulmonary tuberculosis). While sometimes people may exhibit inactive (latent) TB (germs are present, but the immune system prevents them from multiplying), the illness may get active without proper treatment. As such, early and reliable detection of the presence of the infection becomes critical to saving lives.

A typical evaluation for TB starts with the medical history of the patient and a physical examination. Further blood (interferon gamma release assay (IGRA)) or skin (tuberculin skin test (TST) or antigen-based skin test (TBST)) analyses may reveal if the subject has been infected [8]. Moreover, screening methods may be indicative if additional investigations are needed. As such, the most recent guidelines published by the World Health Organization recommend Xpert MTB/RIF Ultra and Truenat assays as rapid diagnostic solutions [8]. Those may be complemented by lab tests on sputum and lung fluid, chest X-ray, or computed tomography (CT) scans.

The recent COVID-19 pandemic triggered an immense research effort targeting the identification of proper sensory data/biomarkers indicative of the presence of pulmonary infections and associated analysis methods and equipment. A plethora of solutions have been proposed and evaluated, mostly involving medical imaging (X-ray, CT, or ultrasound), further analyzed with various deep learning models. Nevertheless, such approaches require highly skilled medical experts, who may still offer different interpretations of the imaging data. Moreover, those solutions involve an expensive testing infrastructure, which is problematic in resource-constrained environments. Since such techniques are costly, non-portable, and may use ionizing radiation, more affordable and less intrusive alternatives have been sought.

TB detection using AI/ML techniques has been the subject of several comprehensive review studies that identify the recent trends and compile state-of-the-art performance metrics [9,10]. Most of the methods use chest radiographs (CXR) or CT scans datasets, building on the remarkable classification performances of convolutional neural networks (CNNs) and visual transformer models. Modern solutions include multi-modal input sources, integrating imaging data, laboratory/clinical information, and narrative unstructured data, paving the way for the use of vision-language learning models. Many studies incorporate explainable AI (XAI) approaches, yielding transparency on the operating mode of the various architectures and enhancing trust in the diagnosis outcome. One key aspect involves coping with the limited data availability or heavily unbalanced datasets scenarios, which is tackled by considering synthesis solutions based on the SMOTE method [11] or various generative algorithms. Topics that need further in-depth analysis are related to reliable discrimination between latent and active TB (since the former does not present visible imaging abnormalities), and the systematic study of the domain-shift problem when using the transfer learning approach (reliable classification of external test datasets that may exhibit different distributions as compared to the data the models were trained on).

One active line of research considers non-semantic health acoustics for detecting various respiratory maladies, including bronchitis, pertussis, chronic obstructive pulmonary disease, pneumonia, COVID-19, or TB. The approaches use audio-type data (cough, speech, or breath) as a potential data source for reliable pneumological infection detection. Cough sounds possess a discriminative potential since previous research showed that the glottis behavior is highly dependent on the pathological condition of the subjects [12]. Nevertheless, discriminating between TB and other pulmonary illnesses is very challenging, given that the human ear cannot reliably differentiate them. By employing an attention-based mechanism, reference [13] identified the spectral and temporal intervals of a cough episode responsible for classification performance. The study revealed that the initial (high-power, large bandwidth) bursts of energy are essential for TB detection, and this segment of the cough sound originates from the lung itself [12].

The limitations, the sources of bias, the type of extracted features, and the comparative performances of audio-based approaches against standard clinical evaluation have been the subject of intense debate, especially in relation to the COVID-19 pandemic [14,15]. Nevertheless, cough analysis may represent a natural choice for pulmonary TB detection, since persistent cough is one of the main symptoms that is clearly influenced by the anatomical modifications induced by the disease. Moreover, a set of recording applications is available, such as Hyfe Research, AI4COVID-19, and ResAppDx36–38 [16], which have been used to generate cough sound datasets, some of which are publicly accessible.

The list of features extracted from the raw audio recordings varies to a large extent and includes Mel-Frequency Cepstral Coefficients (MFCC), log-filterbank energies, zero-crossing rate (ZCR), and kurtosis, among others. Those are applied as inputs to a broad range of classification models such as logistic regression, Support Vector Machines (SVMs), decision trees, multilayer perceptrons, or XGBoost [17]. Many solutions build on the remarkable classification performances of convolutional neural networks (CNNs), typically operating on the bi-dimensional representations of the raw cough recordings time series as Mel spectrograms or wavelet-based scalograms [18]. A recently introduced transformer-based model, termed DMRNet, including multi-head self-attention layers, showed improved performance over classical CNN architectures [19]. Various NLP-style cough embedding procedures complemented by LSTM or SVM classifiers showed high detection performances, although generally evaluated on small-dimensional datasets [20]. Combining capsule networks with fully connected neural networks for analyzing audio spectrograms showed improved performance over classical CNN models [21]. Recurrent models such as bidirectional long short-term memory network (BiLSTM) and BiLSTM with attention have proved efficient in learning patient invariant features [13].

Most of the existing learning-based approaches use supervised learning algorithms, which result in limited generalization performances in the case of novel tasks and/or out-of-distribution scenarios. A self-supervised learning approach recently introduced in [22], trained on a large and diversified dataset, exhibited top performances on 14 distinct cough inference tasks, including TB detection.

It is worth mentioning that some of the existing approaches use various forms of data augmentation (speed perturbation, pitch shifting, or noise addition) to balance the number of data samples from the TB/non-TB classes. Despite this option, complemented by generative methods such as SMOTE [11], many papers report performances evaluated on small datasets, while very few perform external test set validation.

The present paper proposes the use of Bag-of-Words (BoW) classification models to discriminate between TB-infected and non-TB patients, building on the previous successful application of this approach to COVID-19 detection and ECG-based biometrics [23,24]. An ablation study assesses the effect of using both independent and combined distinct feature extraction procedures and encoding strategies. Performances are evaluated in terms of standard metrics such as the Area Under Curve (AUC), accuracy, sensitivity, and F1-score. Experiments were conducted on two distinct large datasets, using both the original recordings and extended versions obtained by augmentation techniques. Performances were assessed by repeated *k*-fold cross-validation and by employing external datasets. Experimental results indicate that the solution compares favorably with more sophisticated approaches, while accommodating variable-length audio recordings and showing robustness against setup parameters.

Section 2 presents the general architecture of the proposed model, details the components of the BoW classifier, and describes the feature extraction and fusion procedures. Comparative performances against other solutions are reported in Section 3, including the description of the training datasets and the augmentation methods, while topics for further study are finally outlined.

## 2. Bag-of-Words Classification Models for Tuberculosis Detection

**Figure 1:**
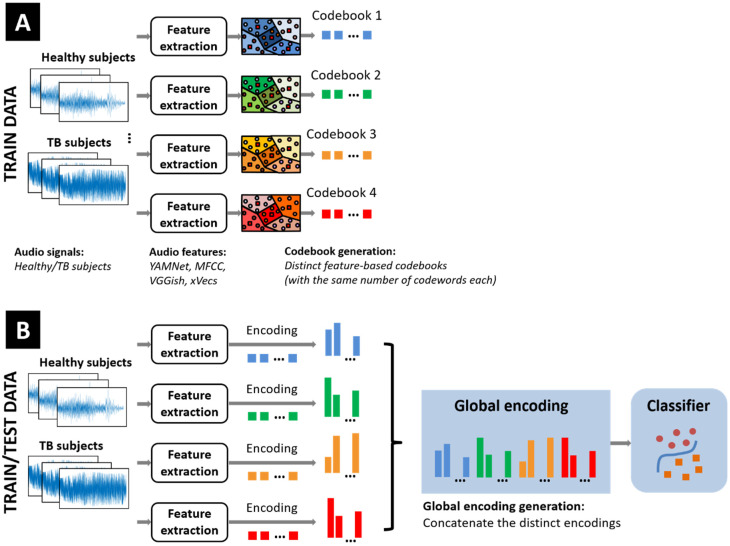
Block diagram of the proposed approach: (**A**) distinct feature-based codebooks generation; (**B**) fusion of individual feature encodings followed by classification.

The block diagram of the proposed approach is indicated in Figure 1. It depicts the components of the BoW classification model and describes the input fusion strategy of the encodings corresponding to the various feature extraction procedures. We describe below the data preprocessing steps, the algorithmic options for implementing each of the constituent modules, and the corresponding setup parameters to be considered in the ablation study reported in Section 3.

### 2.1. Overview of BoW Models

The BoW classification model was originally inspired by text document analysis. Intuitively, we may compactly represent a text by first counting the frequency of appearance of the distinct words composing the text and further plotting the corresponding histogram. This type of representation is agnostic of the order in which the words appear, nor of grammar rules. The approach has been used both for time series analysis [25,26] and computer vision applications [27]. Many options are available for implementing the constituent modules and the associated setup parameters, requiring an in-depth investigation of their optimal values.

In the case of time series analysis, the BoW processing pipeline starts by first pre-processing the raw data, aiming at improving its quality by noise removal and amplitude/time length/sampling frequency normalization. For recordings involving repeated cough sequences, segmentation of the original waveforms into individual cough bursts may also prove beneficial. The following steps are included [23,24]:

(a) Computation of specific (typically, hand-crafted) feature vectors extracted from successive (fixed-length) temporal intervals from the time series under study. In case several distinct datasets are used (e.g., for external set performance validation), all data follow a similar processing procedure, using identical setup parameters.
(b) A set of prototype vectors representative of the feature set distribution is further generated. Those form a codebook including an application-specific number of codewords, typically obtained by employing various clustering algorithms.
(c) A single or, more generally, a combination of specific codewords is next assigned to each feature vector. Special properties of the selected codewords may be imposed by the various encoding algorithms introduced in the literature (e.g., seeking the sparsest subset of codewords that approximates a given feature vector).
(d) Counting the frequency of codeword appearances and computing the corresponding histogram provides a compact description of a given time series. One of the key advantages of BoW models is that the approach can accommodate variable-length time series. As such, the resulting histograms may exhibit variable dynamic ranges, hence the need for using scale-normalization procedures.
(e) The final classification step may consider various models and specific distance measures, some of which are particularly useful when dealing with histogram-type data [28].

The next paragraphs present the design options for each module of the BoW classifier, along the lines of similar setups used in other biomedical applications [23,24].

### 2.2. Data Processing and Feature Extraction

The cough recordings used in the experiments may originate from various sources and exhibit specific temporal and spectral characteristics. As a consequence, we first resample them to a common sampling frequency, apply low-pass filtering, and normalize the amplitude in the [−1, 1] dynamic range. The ablation study presented in Section 3 includes the effect of the sampling frequency and the frequency range on the classification performances.

Non-semantic health acoustics has considered a broad set of features, which may be further subject to various selection procedures such as Local Interpretable Model-agnostic Explanations (LIME) [29] or Principal Components Analysis (PCA). The open-source openSMILE toolkit [30] and Librosa package [31] have been mainly used for computing those features, sometimes complemented by associated statistical functionals (extreme values, energy).

Previous research revealed that the human ear cannot discriminate between TB and COVID-19 cough sounds. As such, the same feature types previously considered for COVID-19 detection [23] have been used in the present paper, all computed from successive (fixed-length) temporal segments extracted from the time series under study:

(a) Mel-frequency spectrogram coefficients computed from 50% overlapping 1 s long audio segments. Distinct spectrograms were generated for each segment with a window size of 25 ms, a window hop of 10 ms, and a periodic Hanning window. A total of 64 Mel bins covering the frequency range from 50 Hz to 4 kHz were used, and after converting the mel-spectrogram into a log scale, we obtained 64 × 96 images per segment. The distinct spectrograms originating from multiple cough bursts acquired from the same human subject were concatenated along the mel band dimension.
(b) Two additional feature types are obtained by intercepting the outputs of specific inner layers of a couple of (pre-trained) convolutional neural network models frequently used in audio applications, the input of which is given by the mel spectrograms described above. The first option is the YAMNet model [32], which yields 1024-long feature vectors by reading the output of the last layer before the classification module (the layer is called global_average_pooling2d in MATLAB R2023b). The model has been pre-trained to identify 521 distinct audio classes, including cough, using the AudioSet-YouTube corpus [32]. As such, it has also been considered a viable solution for segmenting audio recordings and eliminating pauses between actual cough bursts. The second option considers the VGGish model (inspired by the well-known VGG-type image classification architectures) [33], by reading the output of the EmbeddingBatch layer that returns a set of 128-long feature vectors.
(c) *x*-vectors have emerged as a performant speaker identification approach [34], but have also been successfully used in various extra-linguistic tasks. The vectors are computed from successive 1 s long audio segments and a window hop of 0.1 s, extracted from the output of the first fully connected layer of the pre-trained model described in [34]. The resulting 512-long vectors are further reduced to a 150-long common length by linear projection using a pre-trained linear discriminant analysis matrix [34].

### 2.3. Codebook Generation

The generation of the codebooks has usually been approached by considering clustering procedures. For example, given a collection of training data points, the classical unsupervised *k*-means algorithm aims at partitioning the data space into distinct regions and identifying a set of prototype vectors (cluster centers) such that the sum of distances from the data points to the nearest cluster centers is minimized [35]. The Euclidean distance is typically used, while the *k*-medians alternative based on the L1 distance may prove more robust in the presence of outliers. It is worth mentioning that the initialization method critically influences the quality of the clustering procedure. We have used the recommended *kmeans*++ initialization method [36].

More recently, an alternative solution rooted in linear representations over redundant bases has gained much attention [37] and has been successfully used within the BoW framework [23,24]. The method represents feature vectors as a linear combination of a few columns of a dictionary matrix, selected from a set of possible candidates that is much larger than the dimensionality of the vectors under study. Data-independent dictionary options are described in the literature, while data-dependent learning procedures may enable the selection of dictionary components better adapted to the signals of interest [38]. We have used a computationally efficient online training algorithm [39] that updates the dictionary as new data becomes available.

### 2.4. Encoding Procedure

We may choose between two key encoding procedures: hard assigning a feature vector to a single codeword or soft assigning it to a (weighted) combination of multiple codewords. The rationale behind the latter option is that hard assignment suffers from two drawbacks: firstly, a feature vector may be almost equally close to two or more codewords, and still a single codeword is to be selected. Secondly, one codeword has to be selected anyway, although it may be situated far away from the feature vector.

Much similar to previous works [23,24], we consider a collection of *M*-dimensional local feature descriptors $X\;\;=\;\;\left[x_{1},\;x_{2},\;\ldots \;x_{N}\right]\;\;\in \;{ℜ}^{M\times N}$ and a codebook of *K* codewords of the same dimensionality $D\;\;=\;\;\left[d_{1},\;d_{2},\;\ldots ,\;d_{K}\right]\;\;\in \;{ℜ}^{M\times K}$. We define the code of an input vector **x**_i_ as a *K*-dimensional vector **u**_i_ having one or more non-zero entries to accommodate both hard and soft assigning encoding.

*Vector Quantization* (VQ) has been the preferred choice for hard assignment, typically following a codebook generation procedure based on the *k*-means clustering algorithm (or one of its variants) [35]. The encoding is given by:

(1) $$u_{ij}\;\;=\;\;\;\left\{\begin{array}{l} 1,\;\;if\;\;j\;\;=\;\;\arg\underset{j\;=\;1\ldots K\;}{\min}{\left\|x_{i}\;-\;d_{j}\right\|}^{2}\\ 0\;,\;\;\;otherwise \end{array}\right.$$

*Local Linear Encoding* (LLC) minimizes the L2-norm of representing the input data using the given codebook, additionally imposing that the subset of the used codewords should be selected amongst the closer ones in terms of Euclidean distance [40]. LLC provides an analytical solution to the following optimization problem:

(2) $$\begin{array}{l} u_{n}\;\;=\;\;\arg\underset{i\;=\;1\ldots K\;}{\min}{\left\|x_{i}\;-\;Du\right\|}^{2}\;\;+\;\;\lambda \;\left\|s_{n}\;⊗\;\;u\right\|\;\\ such\;\;that\;\;1^{T}\cdot \;u_{n}\;\;=\;\;1\\ where\;\;s_{n}\;\;=\;\;\exp\;\left(\frac{dist\;(x_{n},\;D)}{\sigma }\right) \end{array}$$

*Sparse coding* (SC) seeks the sparsest linear combination of codewords that exactly represents **x** and solves the following optimization problem [37,38]:

(3) $$\underset{u}{\min}\;{\left\|u\right\|}_{0}\;\;such\;\;that\;\;x\;=\;Du$$

where the L0-norm counts the non-zero elements of vector **x**. Since the optimization problem above is computationally intractable, many available approaches replace the L0-norm with the L1-norm [37], leading to a convex alternative that may be efficiently solved. Interpreting the sparsity constraint as a penalty term, the optimization problem above may be recast into a (convex) Lagrangian formulation as

(4) $$\underset{u}{\min}\;\;\;\left\{\;{\left\|x\;-\;Du\right\|}_{2}\;\;+\;\lambda {\left\|u\right\|}_{1}\;\right\}$$

where the optimal value of the *λ* parameter depends on the noise power and the cardinality of the dictionary [41]. The first term is a data fidelity measure and forces the representation **Du** to approximate vector **x**, while the second is a regularization term that reflects a priori knowledge about the given task.

### 2.5. Similarity Measures

While the distance metric measuring the similarity between a pair of vectors is typically chosen as the Euclidean distance, additional classification performances may be gained when dealing with histogram-type data if particular metrics are used instead. Two typical choices are represented by the histogram intersection (*HI*) and chi-squared distances ($\chi^{2}$), respectively, defined as [28]:

(5) $$\begin{array}{l} D_{\chi_{2}}(p,\;\;q)\;\;=\;\;\sum_{k}\;\frac{{\left\|\;p[k]\;-\;q[k]\;\right\|}^{2}}{p[k]\;+\;q[k]\;+\;\varepsilon }\;\;\;\;\;\;\;\;\;\;\;\;\;\;(\chi^{2})\\ D_{HI}(p,\;\;q)\;\;=\;\;1\;-\;\sum_{k}\max\;\left(p[k],\;\;q[k]\right)\;\;\;\;\;(HI) \end{array}$$

### 2.6. Classifiers

A Support Vector Machine (SVM)-type classifier has been used in the experiments to discriminate between healthy and TB-infected people. An RBF kernel of the form $K(x,x’)\;=\;e^{-\gamma {\left\|x\;-\;x’\right\|}^{2}}$ (where *γ* is a positive scalar parameter) has been chosen to implement the well-known kernel trick that would implicitly map generally non-linearly separable data from the original space into a linearly separable one in a transformed higher-dimensional space. We have used the LIBSVM software tool [42] that includes optimization procedures based on nested cross-validation for selecting the value of the *γ* hyperparameter, while also providing probability estimates for multi-class tasks. The possibility of approximating non-linear SVMs by combining linear ones with explicit feature maps has been demonstrated in [43] with significant training/inference speed improvements.

## 3. Experimental Results

This section presents the results of an ablation study aiming at identifying the effect and optimal setting of the various setup parameters of BoW models used for TB detection. Extensive experiments have been performed on two distinct datasets using both 5-fold cross-validation and external test set evaluation. It is worth noting that the different folds include distinct subsets of human subjects, avoiding data leakage from the training set to the test set. Comparative performances indicate competitive outcomes against previously reported results involving more sophisticated approaches or significantly larger datasets.

### 3.1. Training Datasets

Many of the TB detection methods available in the literature use rather small datasets (less than 100 subjects per class) and lack diversity in terms of demographic features, general health conditions, and acoustic sensor types. Table 1 presents some of the publicly available cough datasets, including TB-infected and non-TB (healthy or affected by other pulmonary diseases) subjects, although some papers have considered private data for the experimental evaluation.

Several approaches make use of both forced (solicited) and unsolicited cough sequences, while rather few employ several distinct types of recording devices. The sampling frequency is typically chosen as 16 kHz or 44.1 kHz, and low-pass filtering may be applied to reduce the bandwidth of the signals. The length of the individual time series may vary from single to multiple cough events and may be subject to pause removal, amplitude/duration normalization, or time series to image conversion.

One key point refers to accommodating the practical situations when the TB and non-TB numbers of available samples are unbalanced. In such cases, augmentation techniques are usually considered, operating in the time or the spectral domains. While (random) resampling may offer a potentially viable solution, the original SMOTE algorithm [30] or one of its variants has typically been used as an alternative [44]. Other options include speed modification, pitch shifting, addind background noise, and random masking of the original time series or the associated spectrograms [45].

The experimental results presented in the next sections have considered 5-fold cross-validation as the evaluation procedure performed on the CODA TB [46] and Sharma [18] datasets, respectively. Since the two classes are heavily unbalanced in the former case, we have used the safe-level version of the SMOTE algorithm [44] to generate additional data. External set validation has also been performed by training the BoW models on the CODA TB set and defining the test set by combining the TB data from the Sharma recordings with healthy people’s cough samples from other sources.

**Table 1:** Cough sound datasets used for tuberculosis detection.

Dataset	No. Subjects	Sensor Type	Sampling Rate	Access	Remarks
Wallacedene [17]	16 TB, 35 non-TB	Condenser microphone	44.1 kHz	private	various numbers of cough events per recording
Brooklyn [47]	17 TB, 21 healthy	Condenser microphone	44.1 kHz	private	controlled indoor booth
CIDRZ [22]	46 TB, 183 non-TB	Variable quality smartphones	192 kHz	private	three single coughs and one sequence of multiple coughs
Swaasa [48]	278 TB, 289 non-TB	smartphones and tablets	44.1 kHz	private	10 s recordings, noise filtering
Xu [19]	141 TB, 152 healthy, 52 other resp. diseases	smartphone	44.1 kHz	private	quiet room, augmentation used
Sharma [18]	103 TB, 46 non-TB	3 microphone types	16 kHz, 44.1 kHz	public	various audio bandwidths
CODA TB DREAM Challenge [46]	297 TB, 808 healthy	smartphones with the Hyfe app	44.1 kHz	upon request	2143 patients across 7 countries, 0.5 s segments
Xu [49]	70 TB, 74 healthy	smartphone	44.1 kHz	public	0.35 s multiple cough events

### 3.2. Effect of the Sampling Frequency

Rather few papers have explicitly addressed the effect of the sampling frequency and the audio bandwidth limitation on the TB detection performances. The notable exception is reference [18], which revealed improved results for higher sampling frequency values, while additionally restricting the audio spectrum to the 50 Hz–4 kHz range. Performances reported in Table 2 confirm previous findings, suggesting optimal settings of 44.1 kHz sampling frequency and 4 kHz as the upper audio limit. Using a higher bandwidth (15 kHz) mildly degrades the outcome, while additionally impeding the use of the Sharma dataset for external validation, since the spectrum of this data is limited to 4 kHz. A paired sample *t*-test (following a Shapiro–Wilk normality test) revealed that there is no significant difference in the mean values of the various performance measures between the 4 kHz and 15 kHz upper frequency experiments (*p* > 0.09 at 5% significance level). Training was performed on the CODA dataset [46], and reported results include both (a balanced subset of 290 samples per class of) the original recordings, and an augmented set of 600 samples per class obtained by using the safe-level version of the SMOTE algorithm [44] (augmentation applied only on the under-represented TB infected class).

### 3.3. Effect of the Feature Set Type

**Figure 2:**
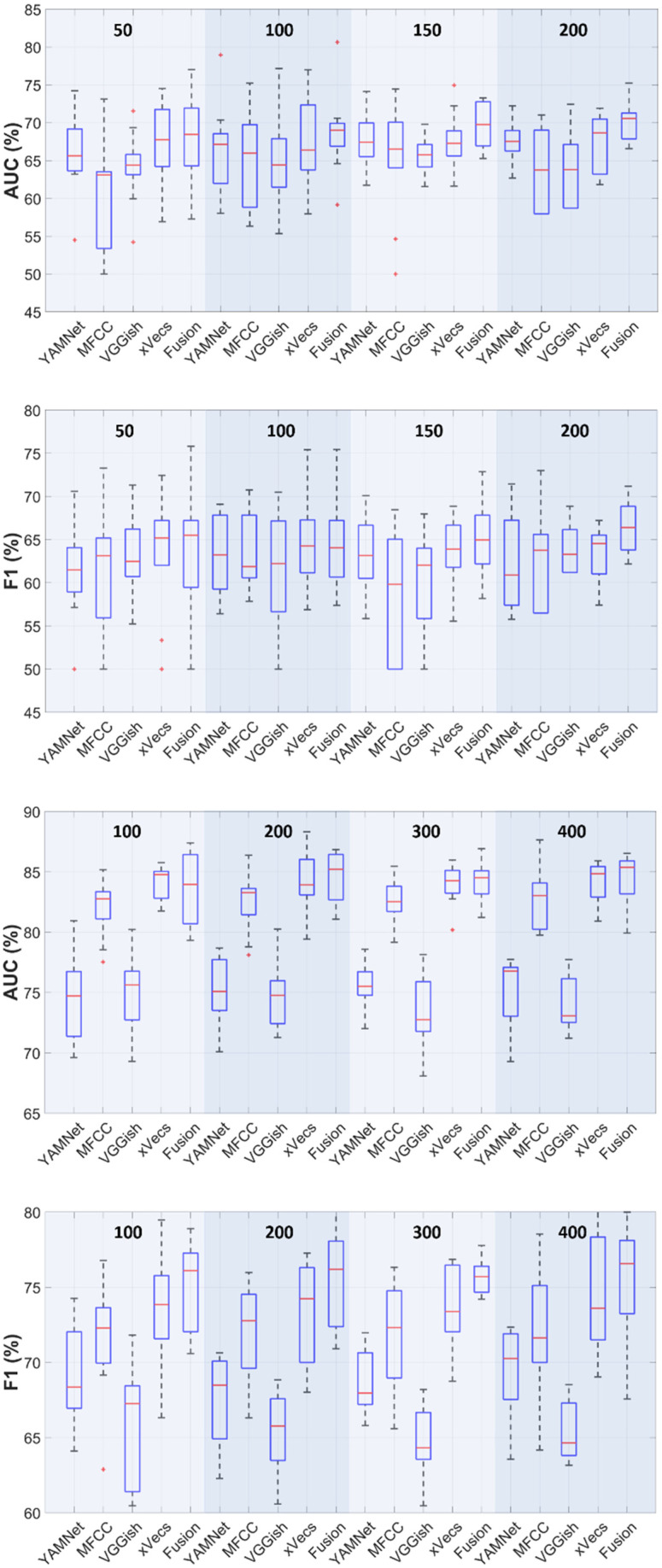
Box plots for AUC and F1 performances vs. the feature set type (sparse encoding). Each boxplot represents the interquartile range (IQR, 25–75% percentile). The center horizontal line represents the median AUC/F1 value. The dashed vertical lines represent the data points within the 1.5 × QR range. Red “+”’s define outliers (AUC/F1 values greater than 1.5 × IQR). Rows 1–2: 290 subjects/class, 50–200 codewords. Rows 3–4: 600 subjects/class using SMOTE, 100–400 codewords.

The effect of the feature set extracted from the audio data is illustrated in Figure 2. Results are indicated in terms of median values, along with the interquartile range (IQR, using the Q1 = 25% and Q3 = 75% percentiles). For 200 codewords and sparse encoding using the non-augmented training set (290 subjects per class), the AUC IQR range varies from [Q1 = 58, Q3 = 69] in the case of MFCC features to [Q1 = 67.8, Q3 = 71.3] for the fusion scenario, while F1 IQR varies from [Q1 = 56.5, Q3 = 65.6] to [Q1 = 63.8, Q3 = 68.8]. The sensitivity results indicate variation from [Q1 = 57, Q3 = 69] for YAMNet features to [Q1 = 63.8, Q3 = 72.4] for the fusion case, while the specificity varies from [Q1 = 58.6, Q3 = 67.2] for *x*-vecs features to [Q1 = 58.6, Q3 = 69] for input fusion. The accuracy performances show a similar behavior, with improved results of the fusion scenario ([Q1 = 63.8, Q3 = 67.2]) over all individual feature sets. The paired *t*-test analysis (following a Shapiro–Wilk normality test) indicated statistically significant differences in terms of accuracy, sensitivity, and specificity between the fusion scenario against the individual use of MFCC features (*p* < 0.01), but not the other options (*p* > 0.12). The F1-score and AUC performances follow the same pattern, regardless of the number of codewords used for encoding. When considering augmenting the dataset by the SMOTE procedure, statistically significant differences appear (*p* < 0.01) only when comparing the fusion approach against the YAMNet and VGGish features.

### 3.4. Effect of the Encoding Procedure

**Figure 3:**
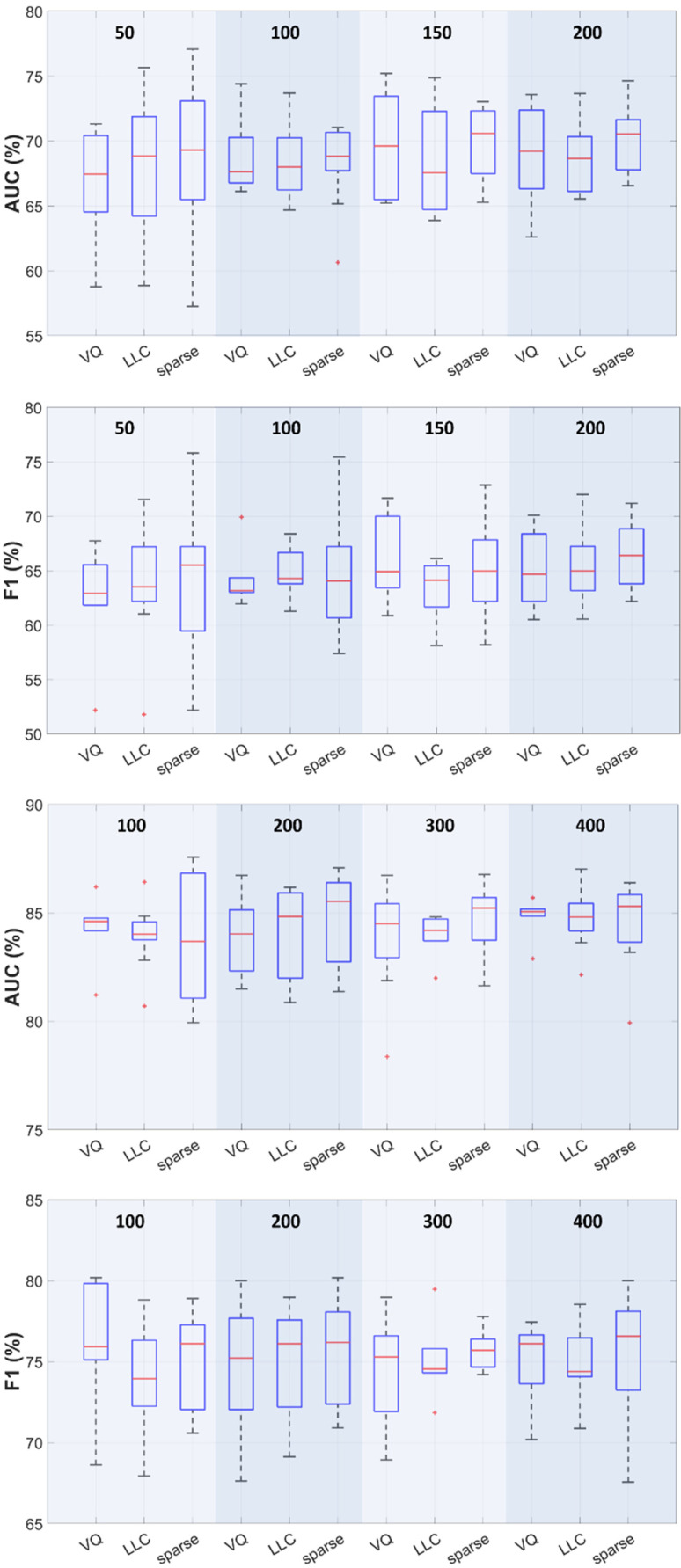
Box plots for AUC and F1 performances vs. the encoding procedure (feature fusion). Each boxplot represents the interquartile range (IQR, 25–75% percentile). The center horizontal line represents the median AUC/F1 value. The dashed vertical lines represent the data points within the 1.5 × IQR range. Red “+”’s define outliers (AUC/F1 values greater than 1.5 × IQR). Rows 1–2: 290 subjects/class, 50–200 codewords. Rows 3–4: 600 subjects/class using SMOTE, 100–400 codewords.

Much similar to the feature type dependence analysis in the section above, the results represented in Figure 3 show a mild performance improvement when increasing the number of codewords. For 200 codewords and fusion scenario using the non-augmented training set (290 subjects per class), the AUC IQR ranges are [Q1 = 66.3, Q3 = 72.4], [Q1 = 66.1, Q3 = 70.3], [Q1 = 67.7, Q3 = 71.6] for VQ, LLC, and sparse encoding, respectively. The corresponding F1 IQR intervals are [Q1 = 62.2, Q3 = 68.4], [Q1 = 63.2, Q3 = 67.2], and [Q1 = 63.8, Q3 = 68.8], respectively. The sensitivity values vary from [Q1 = 63.8, Q3 = 70.7] in case of VQ, to [Q1 = 63.8, Q3 = 69] for LLC, and [Q1 = 63.8, Q3 = 72.4] for sparse encoding, while the specificity ranges are [Q1 = 56.9, Q3 = 63.8], [Q1 = 58.6, Q3 = 65.5], [Q1 = 58.6, Q3 = 69], respectively. The accuracy performances show a similar behavior, with [Q1 = 62, Q3 = 65.5], [Q1 = 62.9, Q3 = 66.4], [Q1 = 63.8, Q3 = 67.2] for the three procedures considered. Nevertheless, the *t*-test analysis revealed no significant differences between the various encoding techniques, regardless of the number of codewords (*p* > 0.06 on any performance measure paired comparison), for both original and augmented datasets.

### 3.5. External Set Validation

The overall best performances using 5-fold cross-validation (based on five repetitions following random resampling of the datasets) are presented in Table 3 for both the CODA and Sharma datasets. Four distinct scenarios were considered: the first two employ original balanced healthy/TB samples for each dataset. The third one augmented the original TB samples from the CODA set with additional data obtained using the safe-level version of the SMOTE algorithm, while keeping the original healthy recordings. The last scenario combined the original CODA and Sharma TB samples, while also using the original CODA healthy data.

While significant, *k*-fold cross-validation results should be complemented by experiments involving external set validation. In fact, very few papers focusing on TB detection use external datasets to validate the performance of the various proposed approaches on data acquired in different recording settings as compared to the training sessions. The differences may consider the type of acoustic sensors, the demographic information, the health status of the human subjects, or the level of background noise.

Table 4 reports external test set results using BoW models obtained in three distinct scenarios: (a) T1: A total of 315 TB subjects from the Sharma dataset [18] and 315 healthy subjects from the Cambridge dataset [50]; (b) T2: A total of 315 TB subjects from the Sharma dataset and 315 healthy subjects from the ComParE dataset [51]; (c) T3: A total of 137 non-TB subjects (presenting pulmonary diseases other than TB, including bacterial pneumonia, viral upper respiratory infection, and asthma) and 137 TB subjects from the Sharma dataset. The original cough recordings for all experiments have been low-pass filtered in the range 50 Hz–4 kHz and use a 44.1 kHz sampling frequency.

**Figure 4:**
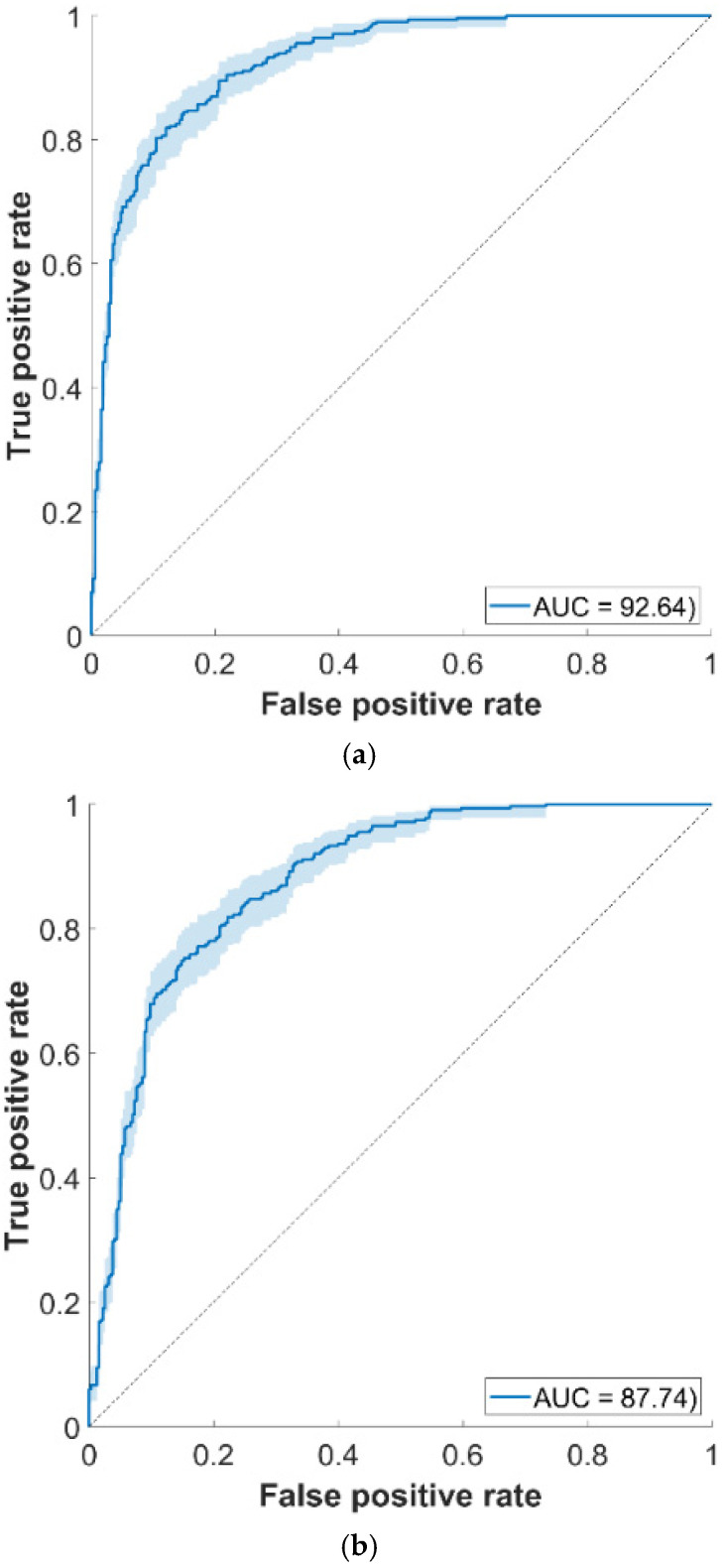
ROC curves for T1 (**a**) and T2 (**b**) external test set scenarios (showing 95% confidence intervals using 1000 bootstrap samples).

The reported results consider input fusion of all feature types and balanced healthy/TB samples for all scenarios. The performances are high for the first two scenarios, as further indicated in Figure 4, while exhibiting much lower values for the third one. Such behavior is to be expected, mainly in terms of reduced specificity, since the non-TB component of the T3 dataset includes subjects affected by various pulmonary diseases, while T1 and T2 contain healthy people, similarly to the CODA TB data used for training the BoW models (290 per class healthy/TB samples). Experiments using the T3 dataset revealed that the individual YAMNet features increase the specificity performances, while the *x*-vecs have an opposite effect, hence the results indicated in Table 4 considered fusing only those two features. Discriminating TB against other pulmonary diseases (instead of healthy status) will be subject to further study.

### 3.6. Comparison Against Other Approaches

Table 5 presents a comparative analysis of the proposed approach against other solutions reported in the literature. While the actual experimental setup may vary to a large extent in terms of the dataset dimension, recording equipment, preprocessing steps, or feature set type, the BoW-based solution exhibits competitive performances against more sophisticated approaches, even on external test sets. Moreover, (the safe-margin variant of) the SMOTE augmentation algorithm clearly improves the results.

Reference [22] uses masked autoencoders trained with a scalable self-supervised learning algorithm and has been evaluated on 33 health acoustic tasks, including 14 cough inference ones. In case of TB detection, the dataset included 24 TB and 240 non-TB human subjects. The masked autoencoder computes low-dimensional 16 × 16 spectrogram patches encoded by a visual transformer model, while the encoded tokens are further processed by an 8-layer transformer decoder. The AUC performance is around 0.74, showing robustness across various recording devices. Reference [52] performed a 5-fold cross-validation training of a logistic regression model using log-spectral energies and MFCC features, on a dataset of 17/21 TB/non-TB subjects. The sequential forward search algorithm enabled the successive selection of the best log-spectral energy features, while output-fusion enabled combining classification decisions made by distinct models trained on audio and clinical data, respectively. Both accuracy and AUC values are placed in the 0.8–0.81 range when using audio (log-spectral energy) data only, while combining audio and clinical information yields an increase in AUC performance (0.95), with a marginal improvement on accuracy (0.82). Reference [17] evaluated the comparative performances of five classification models (logistic regression, SVM, *k*-nearest neighbors, multilayer perceptrons, and CNNs) on a 16/35 TB/non-TB dataset. The feature set includes a total of 78 components, such as MFCC, log-filterbank energies, zero-crossing rate, and statistical moments. Sequential forward selection was used to identify a subset of the best 23 features. Best performances were obtained by the logistic regression model (accuracy = 0.845, AUC = 0.86). The study reported in [48] is one of the few that presents classification results using an external test dataset. The number of TB and non-TB human subjects is rather large (278 and 289, respectively). The total number of original features is 290, further reduced to 170 based on correlation analysis. Accuracy and AUC performances on 10-fold cross-validation were 0.86 and 0.94, respectively, when computed on the original dataset, while reducing to 0.78 and 0.9, respectively, when evaluated on an external dataset including 65 subjects. Reference [21] outperforms most of the available models by combining capsule networks with various CNN architectures. Moreover, the paper proposes an innovative feature set by computing the Histogram of Oriented Gradients (HOG) on audio spectrograms. The hybrid capsule network + CNN models evaluated on the CODA dataset outperformed all individual CNN architectures (VGG16, Resnet-50), yielding an impressive 0.97 performance on both accuracy and AUC measures.

The most relevant comparison should consider the results reported in [46] performed on the CODA dataset. Nevertheless, two significant setup differences may explain the superiority of some of the proposed solutions. The first refers to the actual means of coping with the healthy vs. TB data imbalance. While we have used a variant of the SMOTE algorithm, the best solutions in [46] applied random resampling of the under-represented class. Secondly, besides the solicited (voluntary) cough sequences that have been used in the current report, the top-performers in [46] have also used a large number of unsolicited (reflex) cough recordings (acquired in unsupervised conditions and including only a subset of the participants) that exhibit differences against the voluntary cough sounds [53].

## 4. Discussion and Conclusions

The existing approaches to TB detection vary to a large extent in terms of the data conditioning procedures that may critically impact the reported performances. The controlled (indoor, quiet room) vs. uncontrolled acquisition scenarios, the characteristics of the recording devices, the data/noise filtering procedures, or the sampling rate may have a significant impact on the final outcomes, while creating difficulties for performing fair comparative evaluations.

The results presented in Table 2 confirm the decision to restrict the frequency bandwidth of the cough recordings to the lower audio range (50 Hz–4 kHz), while adopting higher sampling rates (44.1 kHz). When considering data originating from different sources, current practice may require resampling to a common sampling rate, while additionally normalizing the amplitude range.

The effect of the feature types extracted from the (preprocessed) cough signals indicated in Figure 2 and further analyzed using the *t*-test revealed no statistically significant differences among the various choices, while the fusion of the individual encodings and increasing the number of codewords generally results in a mild improvement of the average performance. Figure 3 shows that LLC and sparse coding offer comparable performances, confirming previous results [23,24], while exhibiting robustness against the number of codewords.

Table 3, Table 4 and Table 5 offer a comparative analysis against previously published results. A fair evaluation is limited by the characteristics of the experimental setup in terms of the number and diversity of the cohorts, the acquisition framework, and the specific means of coping with the frequent situation of class imbalance. Only a few papers consider large and demographically variable datasets, multiple microphone types, and external set validation. Nevertheless, accuracy values in the range 65–77% and AUC up to 84% favorably compare the BoW approach with more sophisticated solutions based on various CNN and recurrent deep learning architectures, which typically require large training datasets. Moreover, external test set results in Table 4 are in line with or slightly better than those reported in Table 3, given that the data were compiled from various sources.

One aspect worth mentioning is related to the statistical analysis of the performance results reported in Figure 2 and Figure 3, and Table 3. It is well-known that standard *k*-fold cross-validation may yield high values of so-called Type I errors (erroneously rejecting the null hypothesis and deciding that there is a statistically significant difference between results in cases where there should not be). To remedy this, various forms of corrected *t*-test and the use of repeated *k*-fold cross-validation have been proposed in the literature [54,55]. In our experiments, the 5-fold tests were run five times, the *t*-statistic was computed as in [54], and a 5% significance level was used in all cases.

All experiments were performed in MATLAB 2023b, on a Windows 10 workstation with 64 GB RAM and RTX 3090 GPU. Storing the codebook requires 8.4 kb/codeword, and the encoding procedure uses 2 kB to store a 100-long histogram. The encoding time in case of input fusion is about 0.2 s, while the inference time is 1.23 s for a 2 s long cough audio sample.

After the end of the recent COVID-19 pandemic, tuberculosis has regained its terrible first rank on the list of death toll caused by infections. The disease affects many countries and all age ranges, and is especially aggressive for people with HIV or exhibiting antimicrobial multidrug resistance. If detected early, TB can be cured, although most of the current screening and detection methods require highly skilled staff and costly medical infrastructure. Since financial and technical constraints may be critical in many real-life scenarios, there is a significant interest in finding affordable and user-friendly settings to enable the reliable detection of the illness.

The pandemic has triggered an immense research effort that included the identification of proper markers for discriminating the presence of the COVID-19 infection from other pulmonary diseases or the healthy status of the subjects. Audio features have drawn special attention, since speech, breath, and mainly cough are clearly influenced by the condition of the lungs. While the performances reported in the literature may sometimes seem too optimistic and be affected by various sources of bias, audio recordings still represent a very convenient data source and may provide added value to more sophisticated evaluation methods.

The present paper proposes the use of a well-established classification model based on the Bag-of-Words approach to discriminate between TB and non-TB infected subjects (including healthy ones). The performances are reported for both original and augmented time series, showing competitive results when compared to more resource-demanding approaches. This is also valid for external test set evaluation, which provides a more realistic assessment of the generalization capabilities of the method that may operate in setup scenarios different from the training phase. One of the main limitations of the BoW classification model is that it does not make use of the temporal structure of the cough recordings. Moreover, it has a rather limited capacity of discriminating between TB and other pulmonary illnesses on (external) test sets, given that the training dataset only included TB-infected and healthy human subjects. While this limitation could have been tackled by using generative classification models such as one-class SVMs, experimental evaluation of this option yielded poorer results as compared to the binary classification problem.

Further work may consider a systematic study of the effect of various augmentation techniques on the classification performances. Applying time series-to-image transformations combined with visual BoW models may also prove worth analyzing. The efficiency of the proposed method should also be evaluated within a multi-class task, by considering the detection of multiple pulmonary diseases.

## Figures and Tables

**Table 2:** Dependence of the training classification performances on the sampling rate and frequency range (CODA dataset, fusion sparse encoding, 200/400 codewords for 290/600 subjects per class experiments, average values using 5-fold cross-validation).

Dataset parameters	Subjects per Class	Accuracy	Sensitivity	Specificity	Precision	F1-Score	AUC (CI)
Sampling rate: 16 kHz Filter: 50 Hz–4 kHz	290	63.4 ± 3.8	67.9 ± 7.2	59 ± 6.4	62.4 ± 3.8	64.9 ± 4.2	66.7 (63.1–70.2)
	600	74.2 ± 4.2	61.1 ± 7.7	87.3 ± 3.6	82.8 ± 4.5	70.2 ± 5.8	82.7 (80.7–84.7)
Sampling rate: 44.1 kHz Filter: 50 Hz–4 kHz	290	65.8 ± 2.6	67.9 ± 5.1	63.6 ± 5	65.2 ± 2.8	66.4 ± 3	70.3 (67.1–71.7)
	600	77.2 ± 3.1	70.4 ± 5	84 ± 3	81.5 ± 3.2	75.5 ± 3.7	84.5 (83–86)
Sampling rate: 44.1 kHz Filter: 50 Hz–15 kHz	290	64.6 ± 4.2	66.2 ± 6.4	63.1 ± 5.2	64.7 ± 5.8	65.1 ± 3.7	69.5 (65.8–73.3)
	600	75.7 ± 3.7	63.6 ± 6.9	87.8 ± 2.4	83.9 ± 3	72.2 ± 5.1	84.5 (82–87.3)

**Table 3:** 5-fold cross-validation classification performances (sparse encoding, input fusion, average values ± standard deviations).

Dataset	Subjects per Class	No. CodeWords	Accuracy	Sensitivity	Specificity	Precision	F1-Score	AUC (CI)
Sharma [18]	110	50	75.9 (±6)	74.5 (±6.9)	77.2 (±6.2)	78 (±6.8)	75.7 (±5.1)	83.9 (78.7–89.2)
CODA [46]	290	200	65.8 (±2.7)	67.9 (±5.1)	63.6 (±5)	65.2 (±2.8)	66.4 (±3)	70.3 (67.1–71.7)
CODA + SMOTE	600	400	77.2 (±3.1)	70.4 (±5)	84 (±3)	81.5 (±3.3)	75.5 (±3.7)	84.5 (83–86)
CODA + Sharma	600	400	73.1 (±4.1)	63.8 (±4.6)	82.5 (±5.2)	76.7 (±5.2)	77.2 (±5.5)	83 (81–85)

**Table 4:** External test set classification performances (sparse encoding, input fusion).

Dataset	Subjects per Class	No. Codewords	Accuracy	Sensitivity	Specificity	Precision	F1-Score	AUC
T1	315	200	83.9	78.1	89.8	80	84.8	92.6
T2	315	200	78.1	70.1	85.4	74.3	79.4	87.7
T3 (YAMNet + xVecs)	137	200	56.6	54	59.3	57	55.4	57

**Table 5:** Comparative performances against other approasches.

Reference	Features/Classifier	No. Subjects	Validation	Performances	Remarks
Baur et al. [22]	Self-supervised deep learning/masked autoencoders	24 TB, 240 non-TB	Internal	AUC: 0.739	Robustness against recording devices
G.H.R. Botha et al. [52]	Log-spectral energies + MFCC/Logistic regression	17 TB, 21 healthy	Internal	Accuracy: 0.80/0.63 AUC: 0.81/0.71 for log-spectral energy/MFCC	Sequential forward search used for selecting the best features
CODA TB DREAM Challenge [46]	CNN on spectrograms; Selected features from Librosa library + Gradient Boosting Decision Tree	297 TB, 808 healthy	Internal	AUC: 0.689–0.743 across algorithms	50 Hz–15 kHz audio range, random resampling to cope with data imbalance
M. Pahar et al. [17]	MFCCs, log-filterbank energies, zero-crossing rate, kurtosis/ Logistic regression	16 TB, 35 non-TB	Internal	Accuracy: 0.845 ACC: 0.863 ± 0.06	Sequential forward search for best feature selection
Sharma et al. [18]	CNN on scalograms	103 TB, 46 non-TB	Internal	AUC: 0.61–0.86	Experiments using 3 distinct mic types
G.D. Yellapu et al. [48]	MFCC, spectral, chroma, contrast, statistical moments/CNN, ANN	278 TB, 289 non-TB	External	Accuracy: 0.78 AUC: 0.9	Selected features Explainability using LIME
G. Frost et al. [13]	Mel-spectrograms, Linear filter-bank energies, and MFCC + BiLSTM	28 TB, 46 non-TB	Internal	Accuracy: 0.68–0.8 AUC: 0.769–0.862	Data augmentation
S.J.S. Rajasekar et al. [21]	Spectrograms + HOG features/capsule networks	297 TB, 808 healthy	Internal	Accuracy: 0.89–0.97 AUC: 0.81–0.97	Spectral subtraction to reduce noise
Present paper	MFCC, YAMNet, VGGish, x-Vecs/BoW	up to 600 per class	External	Accuracy: 0.65–0.77 AUC: 0.70–0.84	Data augmentation, input fusion

## Data Availability

Data are contained within the article.
